# Great Britain transport, housing, and employment access datasets for small-area urban area analytics

**DOI:** 10.1016/j.dib.2019.104616

**Published:** 2019-10-14

**Authors:** Obinna C.D. Anejionu, Yeran Sun, Piyushimita (Vonu) Thakuriah, Andrew McHugh, Phillip Mason

**Affiliations:** a Urban Big Data Centre, 7 Lilybank Gardens, University of Glasgow, Glasgow, United Kingdom; b Department of Geoinformatics and Surveying, University of Nigeria, Nsukka, Nigeria; c Rutgers University, Bloustein School of Planning and Public Policy, United States

**Keywords:** Urban area analytics, Public transport accessibility, Housing datasets, Employment and labour market, Small area assessment

## Abstract

This paper provides a brief description of three new forms of key datasets relevant to urban analytics studies namely: Transport, Housing and Employment Accessibility, covering Great Britain, developed by the Urban Big Data Centre (UBDC). Full details of the research related to this paper are contained in “Spatial urban data system: A cloud-enabled big data infrastructure for social and economic urban analytics” [1]. The transport Dataset contains public transport availability (PTA) indicators at both the stop/station and small-area levels (lower layer super output area (LSOA) and middle layer super output area (MSOA)). The employment dataset provides information on the number of people with access to employment within specific distances from each output area. The housing datasets contains quarterly house rent and sales prices aggregated at output area level (MSOA). The theoretical background for measuring the datasets at small area levels is also presented in this paper. Additionally, a variety of raw data used to produce some of the datasets (e.g. PTA) is also included to enable interested readers to reproduce them.

**Table:** Specifications Table

Subject area	Social Science, Urban Studies, Transport Studies, Employment, and Housing
More specific subject area	Urban Area Analytics, Public transport services, Employment Access, and Housing Affordability
Type of data	CSV and Shapefiles
How data was acquired	Commercial listings, Survey, UK census data, UK Ordnance Survey data, Public Transport Schedule Data, and Office of National Statistics
Data format	Raw, Aggregated, Anonymized, Synthetic
Experimental factors	The transport dataset was transformed from TransXchange Format to general transit feed specification (GTFS), API was used to retrieve the housing data before being reprocessed, travel to work from UK Data Service's Flow Data portal was linked to output area spatial boundaries using the geocodes.
Experimental features	New metrics were calculated based on a combination of different data sources. The GTFS data was subsequently used to create the PTA metrics at LSOA and MSOA levels. Census and travel to work datasets from UK Data Service's Flow Data portal were used to create employment access metrics. Housing metrics were computed from Zoopla housing.
Data source location	Great Britain.
Data accessibility	Some of the datasets that are publicly sharable are can be accessed from the Mendeley Data Repository (https://doi.org/10.17632/tvnnb7pv8b.2). Housing data can be accessed from: https://www.ubdc.ac.uk/data-services/data-catalogue/housing-data/zoopla-property-data/. Others that are safeguarded can be obtained from the UBDC data repository (https://www.ubdc.ac.uk/data-services/data-services/access-our-services/)
Related research article	Anejionu, C.D.O., Piyushimita, V.T., McHugh, A., Walpole, R., McArthur, D., Sun, Y., and Phil Mason. (2019). Spatial urban data system: A cloud-enabled big data infrastructure for social and economic urban analytics. Future Generation Computer Systems, 98, September 2019, 456–473. https://doi.org/10.1016/j.future.2019.03.052

**Table:** 

**Value of the Data**
• Data provides country-wide urban area metrics (public transport availability (PTA), Housing, and Employment access) at small-area levels as well as stop/station-level (for PTA, based on service frequency and service area)
• The new urban area metrics can be used to study spatial and social inequalities in various facets of the urban areas (transport access, rental market dynamics, access to jobs, educational deprivation), and further estimate health, job, and educational outcomes of populations living in deprived areas (e.g. poor public transport services) see Anejionu et al. (2019).
• The data can also be used to compare impacts of policies, industrial and structural changes on intra-city dynamics across the entire country
• Data provides increased frequency of assessing and tracking changes in critical aspects of the urban area (housing rent prices fluctuations, spatial inequalities in PTA etc.) compared to decennial census or national survey datasets
• Longitudinal datasets can be used for in monitoring intra- and inter- annual spatiotemporal changes in the urban area with high level of spatial precision

## 1 Data

### 1.1 Transport data

The transport data provide public transport availability indicators at both the stop/station and small area levels across Great Britain (England, Wales and Scotland). Specifically, we provide stop-level public transport availability data (“GB_STOP_PTAI_2016.csv”, “GB_STOP_PTAI_2016.shp”), LSOA-level public transport availability data (“GB_LSOA_PTAI_2016.csv”, “GB_LSOA_PTAI_2016.shp”), and MSOA-level public transport availability data (“GB_MSOA_PTAI_2016.csv” and GB_MSOA_PTAI_2016.shp).

**Fig. 1:**
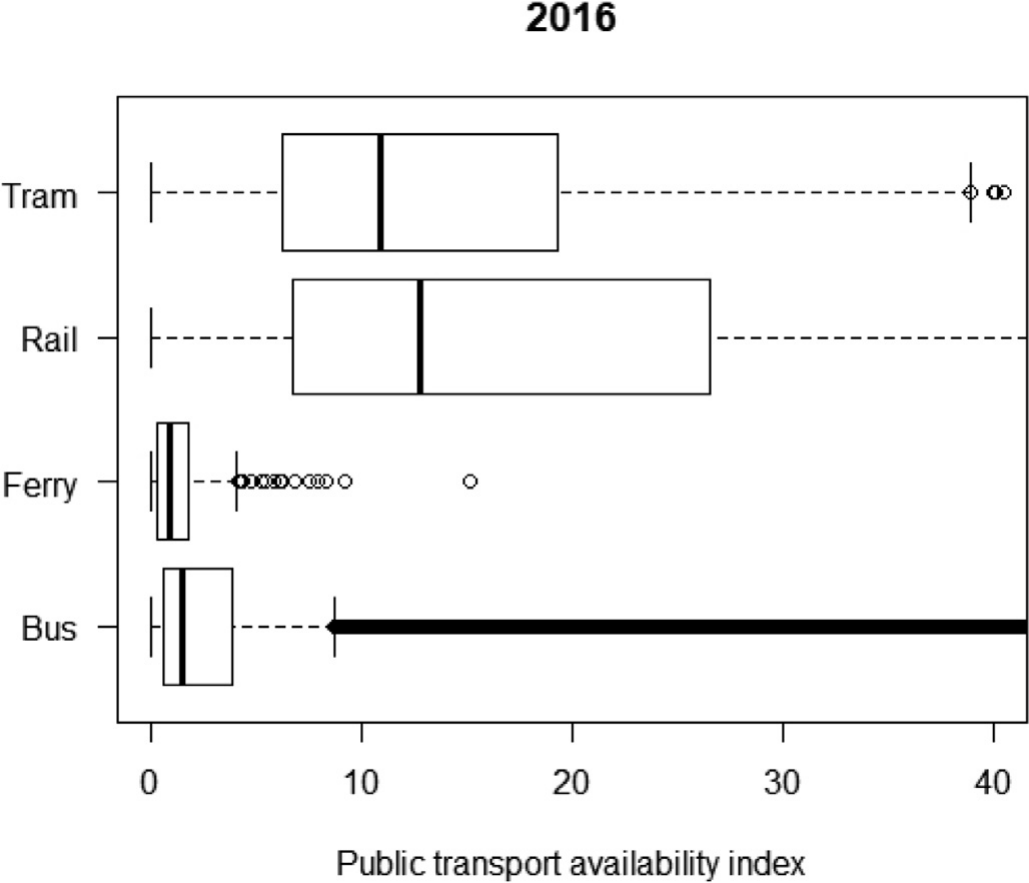
Distribution of stop-level PTAI by public transport service type, 2016.

**Fig. 2:**
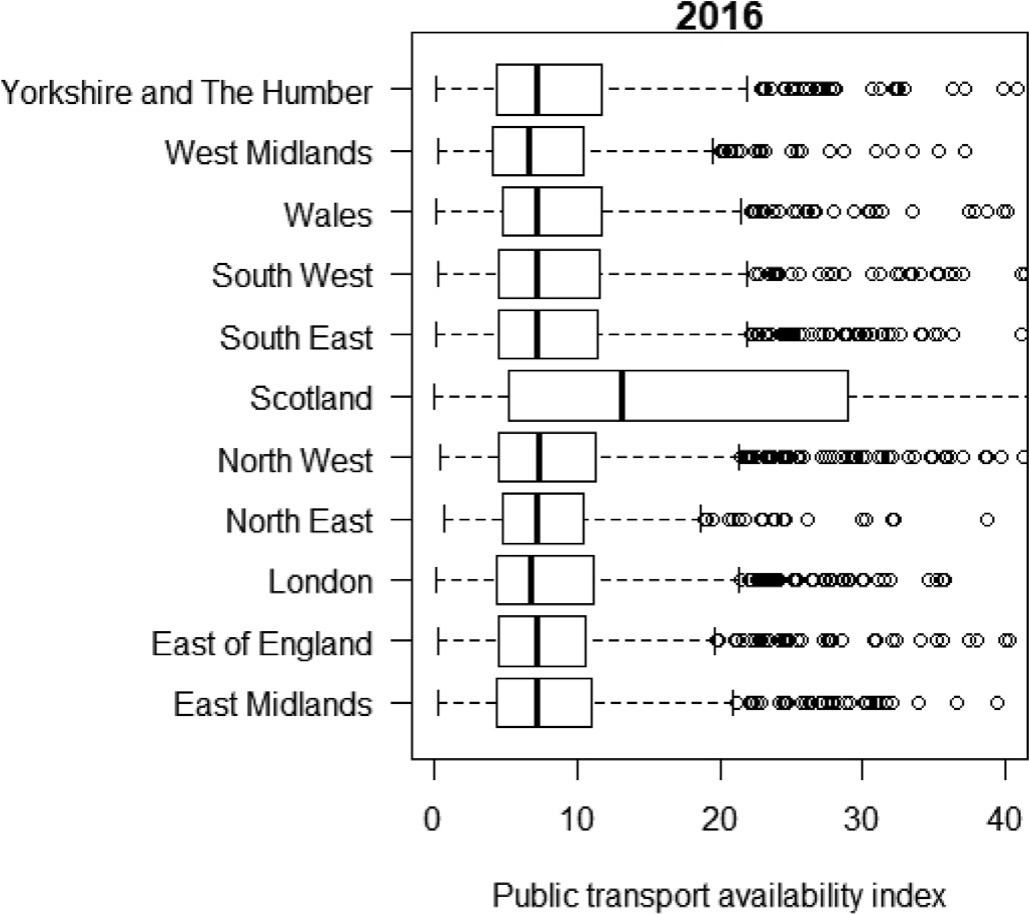
Distribution of MSOA-level PTAI for regions of GB, 2016.

Table 1 shows the number of observations in the public transport availability datasets at both the stop/station and small area levels across Great Britain. Fig. 1 shows the distribution of stop-level PTAI by public transport service type. Table 2 shows small area geographies for different regions across Great Britain. Fig. 2 shows distribution of MSOA-level PTAI for regions of GB. Scotland has a higher median of MSOA-level PTAI than other British regions.

**Table 1:** Description of public transport availability data at stop/station and small area levels.

Data	Count of observations	Data table name
Stop-level public transport availability	33,461	GB_STOP_PTAI_2016
LSOA-level public transport availability	41,729	GB_LSOA_PTAI_2016
MSOA-level public transport availability	8480	GB_MSOA_PTAI_2016

**Table 2:** Description of small area geographies for different regions.

Region	Count of MSOAs	Count of LSOAs	Population
North East	340	1656	2,635,506
North West	924	4497	7,203,775
Yorkshire and the Humber	692	3318	5,411,495
East Midlands	573	2774	4,719,430
West Midlands	735	3487	5,783,410
East of England	736	3614	6,119,230
London	983	4835	8,770,860
South East	1108	5382	9,004,501
South West	700	3281	5,508,645
Wales	410	1909	3,113,150
Scotland	1279	6976	5,404,700

### 1.2 Employment access data

This data contains the number of people within specific distances (5km, 10km, 20km, 25km, 30km, 40km, 50 km, 75km, 100km) from each output area with access to employment. It is provided in this article in two formats: CSV (GB_Employment_Access.csv) and shapefile (GB_Employment_Access.shp).

### 1.3 Housing data

The housing data is an aggregated derivative of a data product acquired under license from *Zoopla Property Group (ZPG) Ltd.* It consists of counts of number of advertisements for rental properties and properties for sale, current and historic median rent and sales of over 27 million residential property records across Great Britain, aggregated at MSOA levels and Broad Rental Market Areas (BRMA) across Great Britain at quarterly intervals.

This is a safeguarded data that cannot be shared openly due to legal conditions attached to the license by the data provider. However, it can be accessed by registered non-commercial researchers, for a certain period. Aggregate data tables are available from UBDC website for personal use only. Individual researchers can access property level data for academic, non-commercial research use if they sign up to a corresponding end-user licence agreement. Interested researchers can contact UBDC directly to access this data.

## 2 Experimental design, materials and methods

### 2.1 Great Britain's small-area geography levels

In the UK demographic datasets, lower layer super output area (LSOA) and middle layer super output area (MSOA) are the two main small-area geography levels. MSOAs are built from groups of contiguous LSOAs. Typically, the average population of MSOAs is 7200; while that of LSOAs is 1500. There are now 34,753 LSOAs and 7201 MSOAs in England and Wales (Office for National Statistics, 2015a). Scotland has independent demographic surveys and uses different names to represent the two small area geography levels. Scottish counterparts of MSOA and LSOA are intermediate zone (IZ) and data zone (DZ). Compared to England and Wales, Scotland is less densely populated. Therefore, IZ and DZ have larger areas but smaller population than MSOA and LSOA respectively. The population of MSOAs is 2500–6000; while that of DZ is 500 to 1000. We merge English and Wales LSOA boundaries with Scottish DZ boundaries into a dataset “GB_LSOA_2011”, and merge English and Wales MSOA boundaries with Scottish IZ boundaries into a dataset “GB_MSOA_2011”.

Data provided in this project are aggregated to these small-area geographies as a way to anonymise them and to make them linkable to other socioeconomic datasets usually presented at these geographic levels.

### 2.2 Transport availability index/metrics

We propose a metric – transport availability index (PTAI) – to represent the levels of public transport service provisions at both stop/station and small area levels. Stop-level PTAI was measured by using public transport schedule data and stop/station location data. This was subsequently aggregated to small-area levels (LSOA and MSOA) in order to ensure PTAI is linkable to socioeconomic data at the same geography level. Specifically, stop-level PTAI was first aggregated to LSOA-level PTAI by overlaying service areas of stops/stations with LSOA boundaries. This was further aggregated to MSOA-level PTAI by weighting LSOA's PTAI with its population. Data sources for this including the LSOA boundaries, MSOA boundaries and LSOA-level population are shown in Table 3.

**Table 3:** Data sources for the raw data used.

Data	Source
Non-train public transport service schedules	Traveline Information Limited [2]
Train service schedules	Network Rail Infrastructure Limited [3]
Locations of stops/stations	Traveline Information Limited [4]
Road network	Ordnance Survey [5]
English and Wales LSOA boundaries	Pope [6]
Scottish DZ Boundaries	Data.gov.uk [7]
English and Wales MOSA boundaries	Office for National Statistics [8]
Scottish IZ Boundaries	Data.gov.uk [9]
English and Wales LSOA-level population	Office for National Statistics [10]
Scottish DZ-level population	National Records of Scotland [11]

#### 2.2.1 Public transport schedule data and stop/station location data

Raw public transport service schedule data of GB is offered by UK Traveline Information Limited and UK Network Rail Infrastructure Limited. More specifically, schedule data of non-train services (bus, light rail, tram, and ferry services) is stored in the TransXchange format, called the ‘Traveline National Dataset (TNDS)’ (Traveline Information Limited, 2016a); whilst schedule data of train services is stored in the common interface format (CIF) format, called ‘GB Rail Network’ (Network Rail Infrastructure Limited, 2014). Compared to TransXchange or CIF, general transit feed specification (GTFS) is a readable and widely used format for public transport schedule data. GTFS data of train services is available (Rail Delivery Group, 2016). However, for schedule data of non-train services were converted from TransXchange to GTFS via a Python conversion tool modified by the Urban Big Data Centre (UBDC) on the basis of an existing conversion tool (Mooney, 2016). This was spatially activated by combined it with stop/station location data offered by UK Traveline Information Limited (Traveline Information Limited, 2016b).

**Fig. 3:**
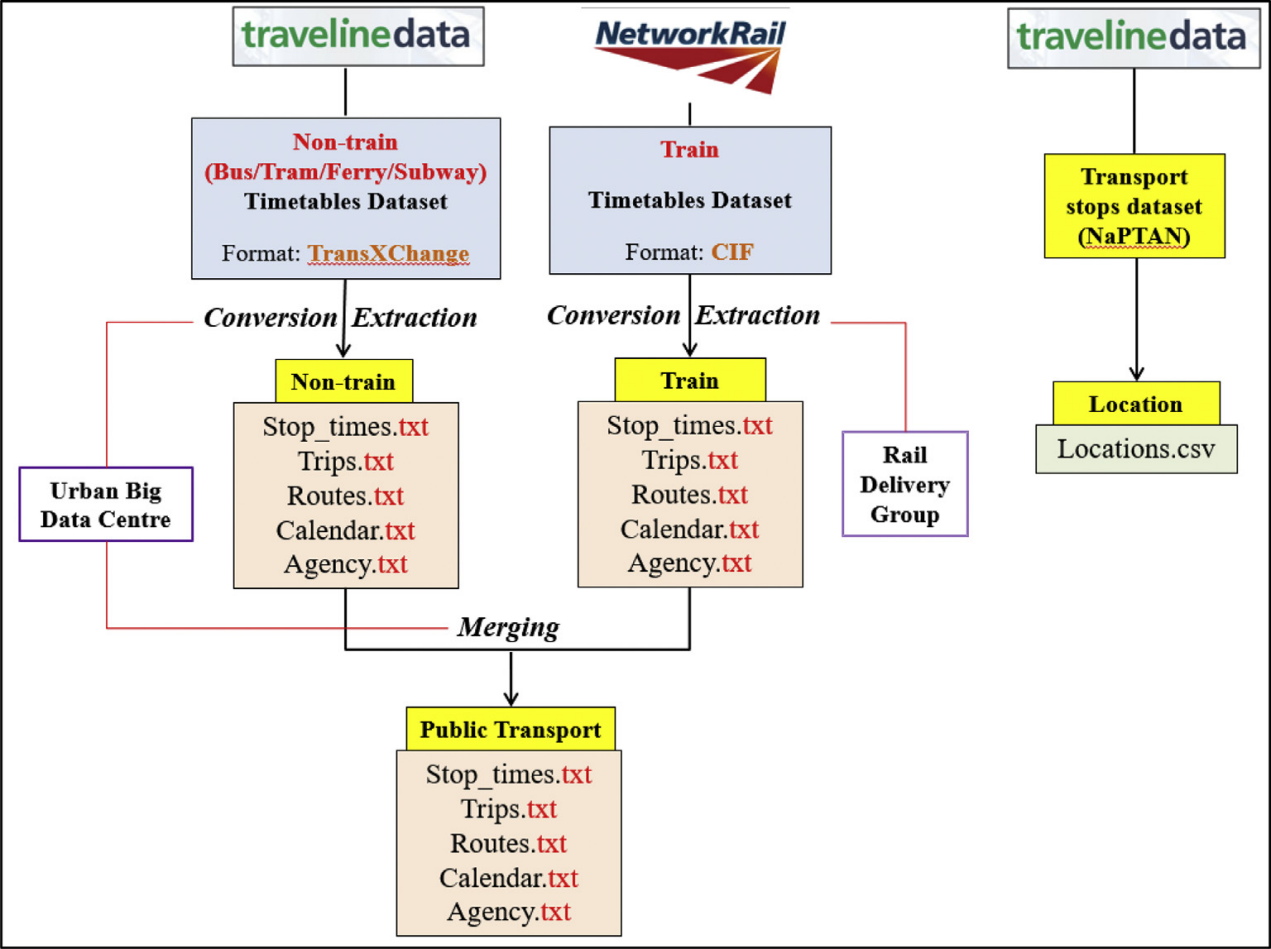
Public transport service schedule data conversion process.

The train and non-train schedule datasets collected in July 2016 were combined into one dataset (“GB_GTFS_2016”) by the UBDC as pilot to demonstrate the generation of this new form of data for accessing public transport availability. Fig. 3 shows the data processing in detail. Based on the GTFS schedule dataset and the stop/station location data collected in October 2016 (“GB_Stop_Location_2016”), 329,314 bus stops, 2514 rail stations, 1325 tram stations, and 306 ferry stations in operation across GB were used. This is in addition to 17,880 bus routes, 5770 rail routes, 93 tram routes and 139 ferry routes in operation.

#### 2.2.2 Stop-level PTAI

To comprehensively measure levels of public transport availability, we take account of service frequency and service area as some studies proposed [[12], [13], [14]]. Moreover, we used an hour-weighted PTAI to represent public transport availability at the stop/station level. Identical service frequency in different daily time periods might influence accessibility for residents differently (e.g., peak time vs off-peak time). Service frequency in peak times seem to play a larger role than that in off-peak times [14]. Specifically, we determine the weights of hourly periods according to hourly distribution of trips in England as Scottish and Wales equivalents are not available. Weights of service hours are proportional to the number of trips in progress within hours as we assumed that high demand of trips within an hour means high importance of the hour. The UK National Travel Survey consists of hourly number of trips in progress on weekdays (Monday to Friday) in England for 2015 [15].

As public transport service schedules differ from weekdays and weekend days, we used only public transport services on weekdays rather than the entire week to measure public transport availability. This is reflective of the fact that vast majority of the residents' journeys to basic destinations such as workplaces and schools occur mostly on weekdays. Hence, the PTA computed here measures how public transport service provisions support basic activities of local residents. Stop-level PTAI was computed as the weighted hourly number of trips passing a stop or station from Monday to Friday. Suppose *i* is a stop/station, its weighted PTAI is calculated as

(1) $$Weighted\_PTAI\left(i\right)=\frac{1}{5}\sum_{t\;\in \;T}cnt\_trip\left(i,t\right)\text{ ∗}w\left(t\right)$$

where cnt_trip(i,t) is the total count of trips passing through the stop (station) *i* during the one-hour period *t* on the five working days, and T is the set of one-hour periods.

#### 2.2.3 LSOA-level PTAI

To accurately and comprehensively measure PTAI at the LSOA level, we took account of both the service levels and service areas of stations/stops. The service area is the area within which people are willing to walk to the station/stop along the road network. The desire to use public transport services declines as walking distance to a bus stop or a train station increases [16]. Some studies reveal acceptable maximum walking distances differ from one public transport mode to another [12,13,16]. A travel survey uncovers that 75%–80% of people would access a stop/station if their walking distances are no longer than mode-specific acceptable maximum walking distances [17]:

• Acceptable maximum walking distance to bus stop = 400 m.
• Acceptable maximum walking distance to tram stop = 400 m.
• Acceptable maximum walking distance to rail station = 800 m.
• Acceptable maximum walking distance to ferry station = 800 m.

A spatial buffer is used to represent service area of station/stop using the respective acceptable maximum walking distances. A circular buffer around the stops/station (Traveline Information Limited, 2016b; see Table 3), and road network buffer, based on the UK Ordnance Survey road network dataset covering Great Britain (see Table 3) [5] were used to generate service areas of stations/stops across GB.

**Fig. 4:**
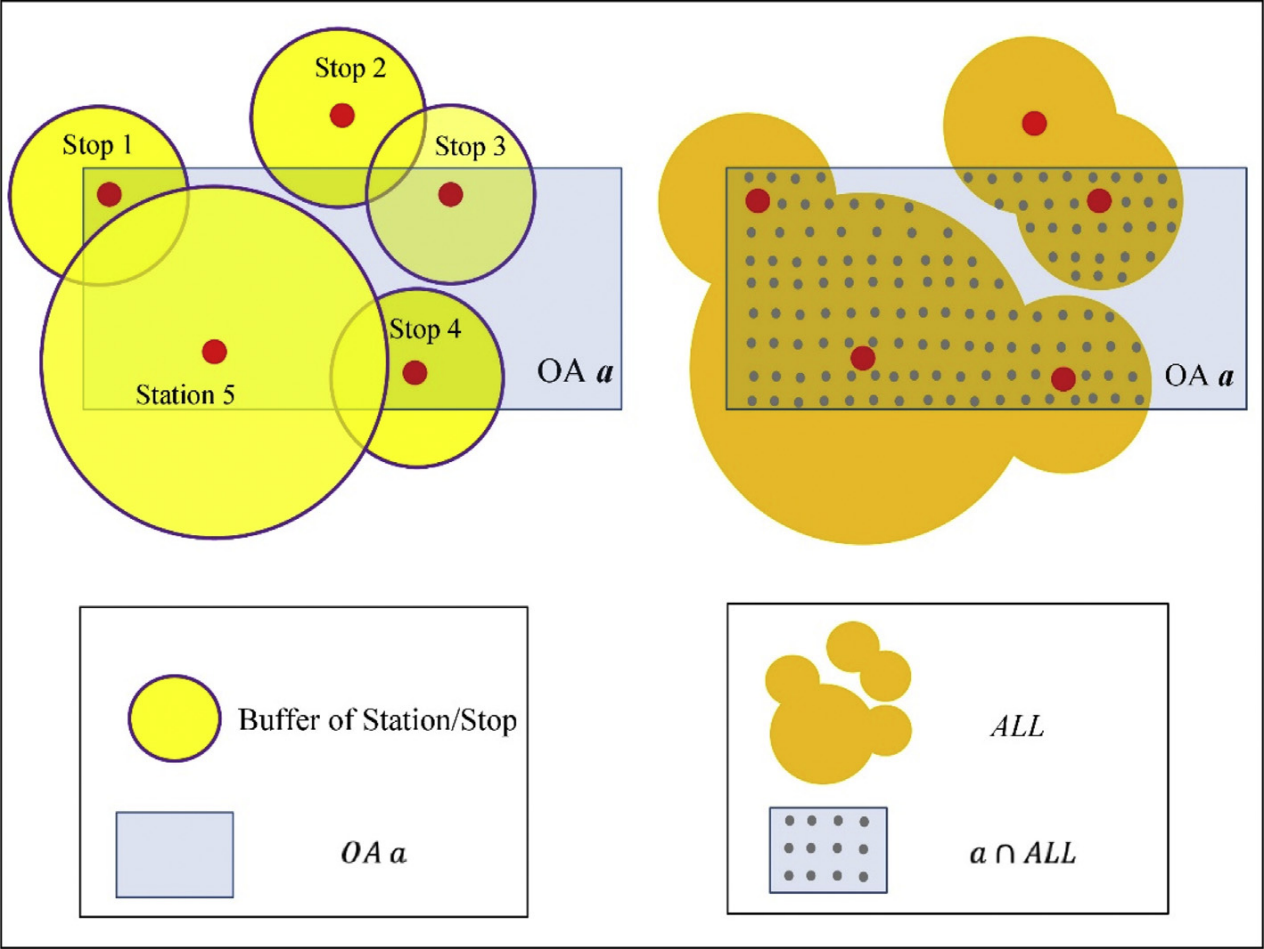
Simplified example of aggregating stop-level PTAI to LSOAs.

Subsequently, stop-level PTAI were aggregated to LSOA by overlapping service areas of stations/stops with LSOAs. Fig. 4 illustrates this, where LSOA *a* is served by Stop 1, Stop 2, Stop 3, Stop 4 and Station 1. For simplicity, regularly shaped buffers (circular buffers) were used to represent irregularly shaped buffers (road network buffers). Part of *a* is not served by any stop/station; while some areas of *a* are served by more than one stop/station. Suppose *L* is a LSOA, its PTAI is calculated as:

(2) $$PTAI\left(L\right)=\sum_{i\in S\left(L\right)}Weighted\_PTAI\left(i\right)\text{*}\frac{Area\;\left(i\;\cap \;L\right)}{Area\left(L\right)}$$

where *i* represents a stations/stop, and S(L) is the set of stations/stops whose buffers intersect *L*. Area(i∩ L) represents the overlapping area between *i* and *L*; and Area(L) is the area of *L*.

#### 2.2.4 MSOA-level PTAI

Population-weighted PTAI was calculated at the MSOA level. Specifically, we aggregated LSOA-level PTAIs to MSOA by weighting LSOA's PTAI with its population. Suppose *M* is a MSOA, its PTAI is calculated as:

(3) $$PTAI\left(M\right)=\;\sum_{j\in S\left(M\right)}PTAI\left(j\right)\text{*}\;\frac{POP\left(j\right)\;}{POP\left(M\right)}$$

where *j* represents a LSOA, and S(M) is the set of LSOAs within *M*. POP(j) is the population of LSOA *j*; and POP(M) is the population of LSOA *M*.

### 2.3 Employment accessibility metrics (EAM)

The need to continuously access more detailed geographical estimates of jobs and locations of workers at small-area levels over time at quarterly, and/or annual intervals motivated the generation of employment accessibility indicators in this project. This is an improvement compared to those currently available from the census or the Office of National Statistics (ONS), which are either aggregated at higher geographic levels (coarser detail) or are available only once every 10 years (decennial). The EAM is expected to enhance the understanding of the performance of different types of jobs (e.g., low-wage jobs or those in the service sector), as the economic dynamics (expansions, recessions or stagnation) changes.

#### 2.3.1 Generation of EAM

**Fig. 5:**
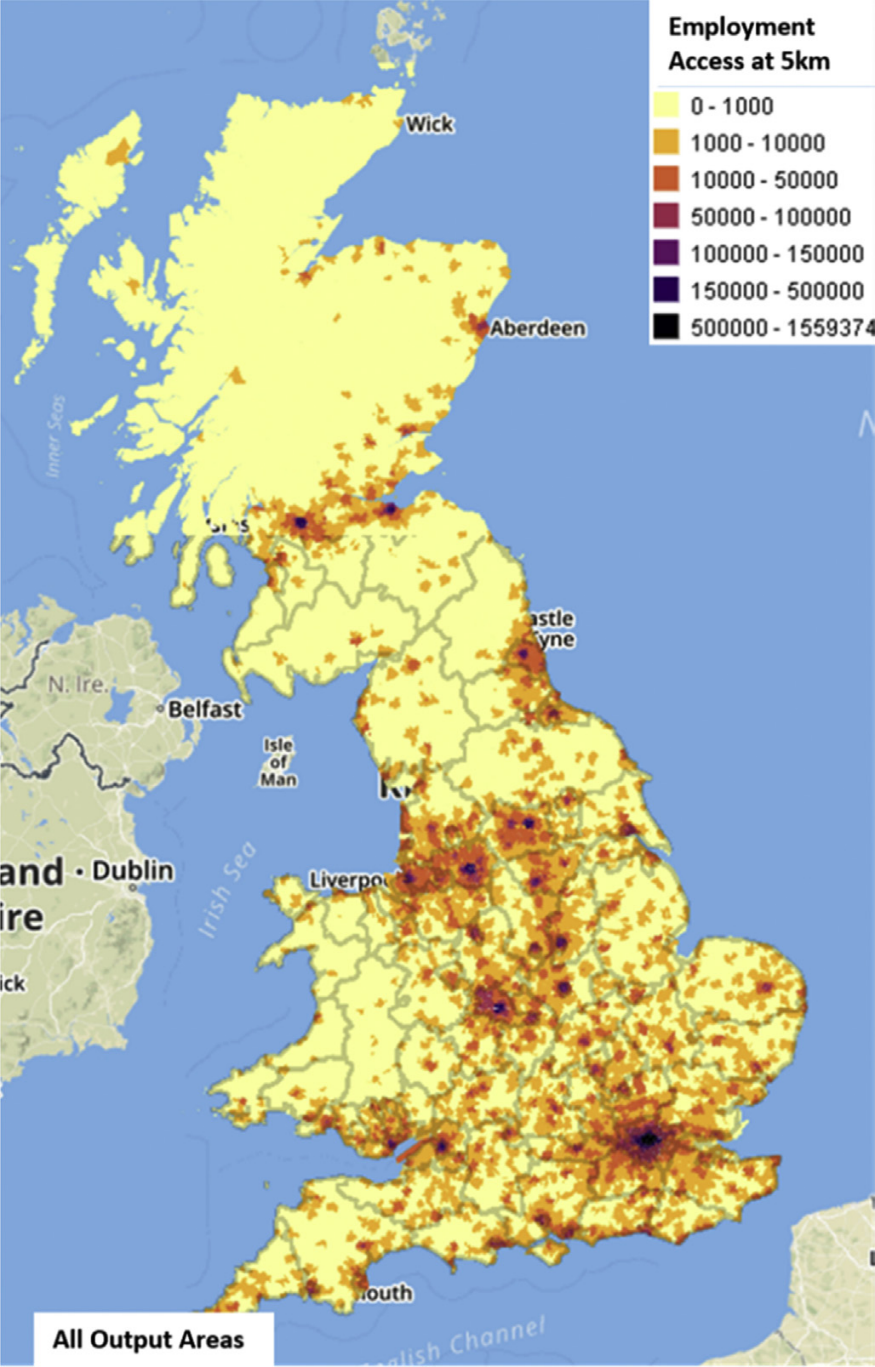
Maps showing employment opportunities within 5km (access 5km) of for all output areas across the GB [1].

The number of people reporting that they worked in each output area (proxy for employment) was derived from travel to work data (2011 census), obtained from the UK Data Service's Flow Data portal. The location of people's residence and work (excluding quasi-workplaces) at the level of output area for the UK, was obtained from Table WF03UK_oa (https://wicid.ukdataservice.ac.uk/). Subsequently, the level of employment in each output area was estimated by aggregating the data by workplace output area. These employment data, combined with travel time information derived from the OpenStreetMap, were used to generate a number of labour market accessibility measures (Fig. 5), using the gravity-based measure of potential accessibility developed by Ref. [18]. A measure of the cost of travelling between each pair of origins and destinations was required in this calculation. Distance along the road network was used as the measure of travel cost. The road network was represented using OpenStreetMap. An all-pairs shortest-path algorithm was then used to estimate a distance matrix.

Different methods have been developed to measure accessibility. A popular gravity-based method developed by Ref. [18] was used to measure accessibility:

(4) $$A_{i}=\;\Sigma D_{j}f\left(c_{ij}\right)$$

where $A_{i}$ is the accessibility index for zone i, $D_{j}$ is a measure of the opportunities available at destination j, $c_{ij}$ is the cost of travel between zones i and j, and f() is a cost deterrence function which reflects how distance affects the accessibility of opportunities. Here, D was used to represent the number of people stating they worked in each output area and $c_{ij}$ will be the network distance between output areas i and j.

The deterrence function was determined using a simple threshold function of the form:

(5) $$f\left(c_{ij}\right)=\;\left\{ \begin{array}{l} 1\;if\;c_{ij}\leq \tau \\ 0\;\;if\;\;c_{ij\;}>\;\tau \end{array}\right.$$

We evaluated the function for different levels of the parameter τ. The accessibility measure gives the number of employment opportunities that can be reached within a given distance. One advantage of this measure is that it is easy to interpret.

### 2.4 Housing affordability metrics (HAM)

Housing indicators are used to highlight the most important features of housing markets [19]. The generation of Housing Affordability Metrics (HAM) in this project was motivated by the considerable knowledge gap concerning the scale and nature of housing dynamics, especially in the UK private-rented sector. The private rented sector is the most dynamic part of the UK housing system, having doubled in size in the last two decades, due to a number of factors including limited mortgage availability and diminished social housing. However, there is little data available to describe the sector [20]. This is due to the fact that most of the available information comes from survey data and decennial census data. Survey data tells a broad story at national, regional and local authority levels, and the UK Valuation Office Agency publishes rent tables to local authority level too. UK Census data provides higher spatial resolution but limited details about the sector. The available data resources are poor at representing lower geographies. This undermines a clear understanding of changes in the sector and associated issues, by local authorities, central government and researchers. Hence, to undertake continuous monitoring of the sector over time, housing market information has to be obtained from alternative sources. Data from Zoopla (https://developer.zoopla.co.uk/), a house listings aggregation service was considered a suitable alternative source for this crucial information. Our aggregate data product derived from the Zoopla property listings website offer additional spatial resolution (at MSOA, BRMA and Local Authority levels), providing details of numbers of adverts and mean/median rents per month by quarter for the period 2011-16. A historical dataset, available for academic, non-commercial research use under EULA terms provides wide-ranging insights about not only the rental and for-sale housing markets but also location, property features and property type within several fields including free text property descriptions and links to associated multimedia content. These have clear and obvious applications for housing researchers but may also be of interest to other urban studies disciplines, or as a corpus or basis for domain application for other data science work, such as text and linguistic analysis.

#### 2.4.1 Zoopla data

Zoopla has over 27 million residential property records in their archive although only a relatively small percentage of these have been advertised for sale or rental on the Zoopla website and therefore contain a property listing history. Zoopla provides access to these historic property listings via an Application Programming Interface (API - https://developer.zoopla.co.uk/docs). UBDC has a licence to access this API with agreement to download data for the UK as part of the Centre's housing data catalogue. Housing data from properties advertised for sale or rent across Great Britain, from 2010 till present, were acquired, and complemented by price paid data (for sales) from the Land Registry of England and Wales and Registers of Scotland.

**Fig. 6:**
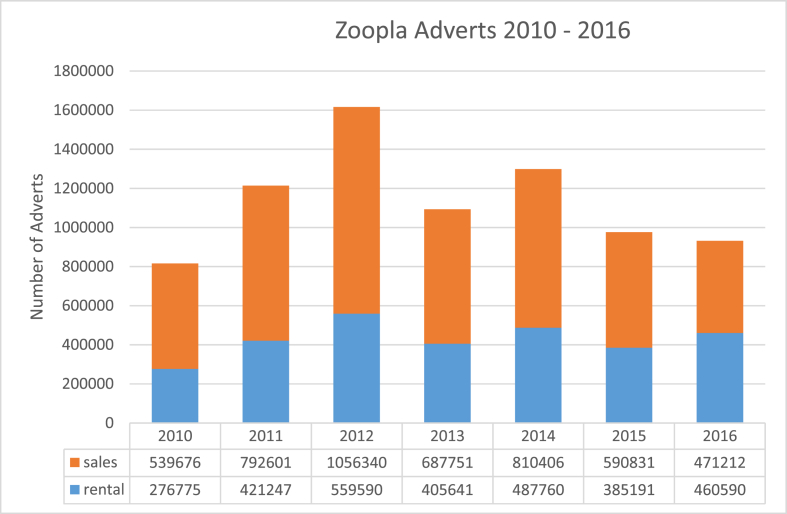
The number of Zoopla adverts by sales and rental for 2010–2016.

Baseline property listings (which contain various types of important historical information about properties) comprising 8 million property records (5 million advertised for sale and 3 million for rent) across Great Britain were initially generated via the Zoopla API with FME data extraction, transformation and loading (ETL) tool, and continuously updated as more properties left the market (closed listings). This has yielded a historic database for GB with over 5 million records of properties advertised for sale and 3 million records of properties advertised for rent. Nightly data collections from Zoopla's live listings API (since August 2016) complement this historical dataset. Full UK coverage is available from 2010 with selected areas from as early as 2005. Fig. 6 shows the number of adverts by sales or rental for 2010–2016, the initial period of historical data collection.

#### 2.4.2 API processing

The Zoopla API request used to retrieve data for individual Zoopla property listing history (https://developer.zoopla.co.uk/docs/Property_listings) requires unique property id. This is included in the active Zoopla property listings, but not in historical datasets. Hence, the Zoopla estimates API, which can use place names, postcode areas or user defined bounding boxes to retrieve individual property information within a specified area, was deployed in retrieving the property ids of historical datasets [21]. To produce the initial property listings for historical datasets (baseline historical dataset) the following steps were taken:

1. Retrieve information of all properties within an area (bounding box) using the Zoopla estimates API (https://api.zoopla.co.uk/api/v1/zoopla_estimates.json?api_key=xxxxxx&lat_min=ymin&lat_max=ymax&lon_min=xmin&lon_max=xmax&page_number=[1-99]&page_size=100)
2. Extract individual property ids from the estimates results set
3. Use extracted property Ids to make API request to retrieve Zoopla property listing history for individual properties (https://api.zoopla.co.uk/api/v1/property_historic_listings.json?api_key=xxxxxx&property_id=nnnnnnnnn)

One kilometer grid (based on the Ordnance Survey's GB grid) that ensured that the whole of GB would be processed as efficiently as possible, was used the area boundary to retrieve property information using the Zoopla Estimates API. The third issue requires the use of an area boundary. The entire process (automated workflow) was setup using the Feature Manipulation Engine (FME), a data integration platform (Extract Transform and Load - ETL tool) developed by SAFE Software.

#### 2.4.3 Housing metrics

A selection of aggregated data tables (Table 4, Table 5) comprising of count of rental adverts per quarter, mean and median rent per month per quarter for Local Authority, Broad Rental Market Area and Middle Super Output Area geographies were produced from the historical dataset. Aggregation to higher geographies was based on postcode so those listings with incomplete postcode information are excluded. Although these tables are available to download with no cost, usage is restricted to non-commercial reference only.

**Table 4:** Mean & median rent per month per quarter by local authority/BRMA/MSOA.

Variable Name	Description
authority_code/area_code	Spatial unit unique identifier
authority_name/brma_name	Spatial unit name
year	4 digit year (2011–2016)
quarter	Quarter of year (1–4)
mean_rent_per_month	GBP mean rent
median_rent_per_month	GBP median rent

**Table 5:** Count of rental adverts by local authority/BRMA/MSOA.

Variable Name	Description
authority_code/area_code	Spatial unit unique identifier
authority_name/brma_name	Spatial unit name
year	4 digit year (2011–2016)
quarter	Quarter of year (1–4)
num_adverts	Total count of rental adverts

**Fig. 7:**
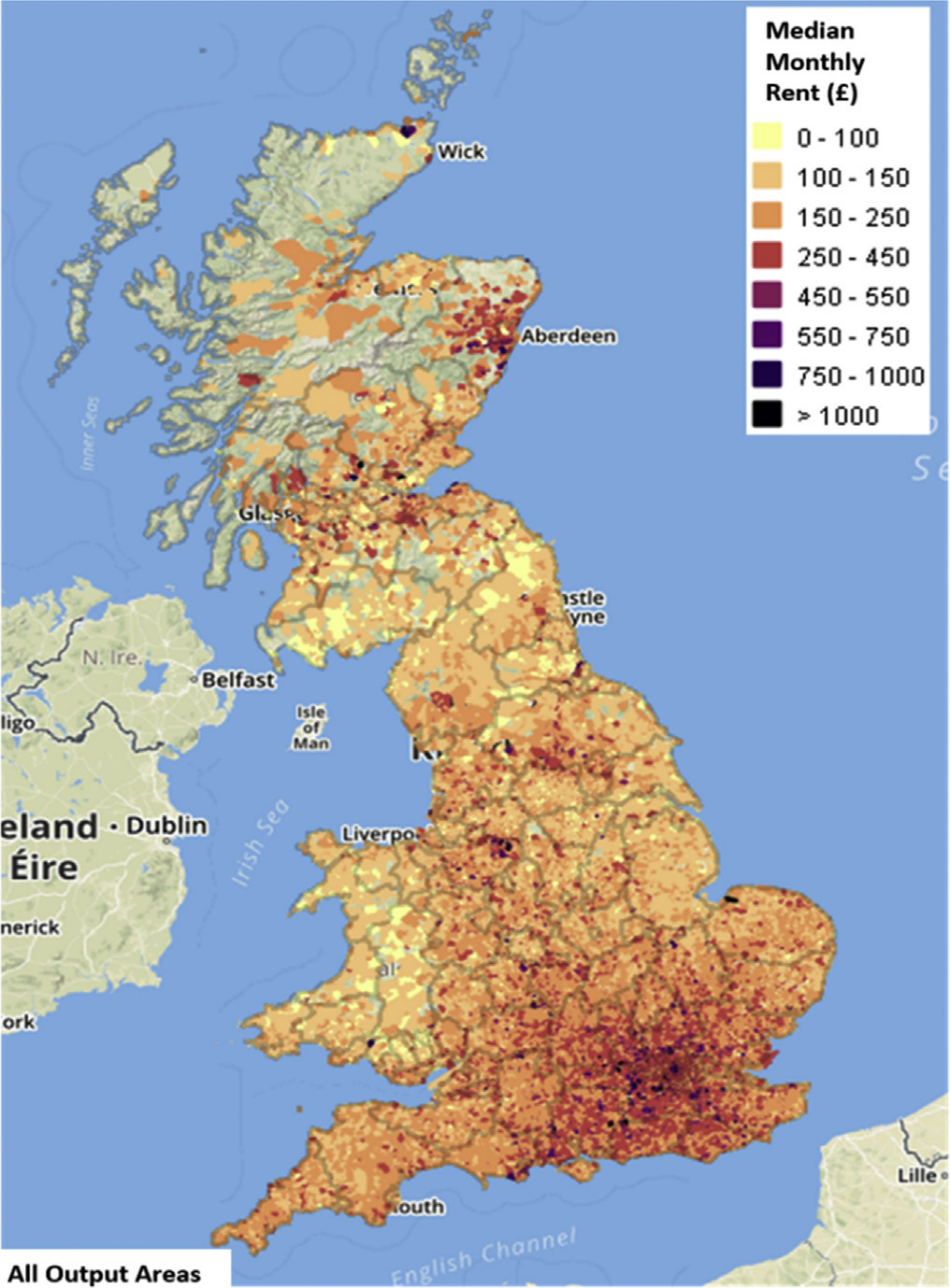
Maps showing monthly median rent price for all output areas across the GB [1].

To generate the housing affordability metrics, relevant housing attributes such as property IDs, address, price, description, date of advert, category, number of floors, were extracted from the Zoopla dataset. The data were linked to the LSOA spatial boundaries through the postcodes. Following this, aggregate data for key statistics (mean, median, maximum price, minimum for the rent and sale prices) of the properties, were computed at LSOA level (Fig. 7).

The housing data can be accessed from the following links.

• Count of number of adverts/quarter by UK Local Authority (XLSX)
• Mean and median rent per month/quarter by UK Local Authority (XLSX)
• Count of number of adverts/quarter by Broad Rental Market Area (BRMA) (XLSX)
• Mean and median rent per month/quarter by Broad Rental Market Area (BRMA) (XLSX)Mean and median rent per month/quarter by Middle Layer Super Output Area (MSOA) (XLSX)
• Count of number of adverts/quarter by Middle Layer Super Output Area (MSOA) (XLSX)
