# Supplementary material for: Great Britain transport, housing, and employment access datasets for small-area urban area analytics
Source: Data Brief. 2019 Oct 14;27:104616. doi: 10.1016/j.dib.2019.104616 (PMC6831819; doi:10.1016/j.dib.2019.104616)
Supplement: Multimedia component 1 [file mmc1.zip › DiB2/Anejionu et al_Data_in_Brief_Manuscript.docx]

**Great Britain Transport, Housing, Employment and Education Datasets for small-area Urban Area Analytics**

Obinna C.D. Anejionu*^^[[1]](#footnote-2)^^, Yeran Sun, Piyushimita (Vonu) Thakuriah, Andrew McHugh, and Phil Mason

Urban Big Data Centre, 7 Lilybank Gardens, University of Glasgow, Glasgow, UK G12 8RZ

# Abstract

This paper provides a brief description of four new forms of key datasets relevant to urban analytics studies namely: Transport, Housing and Employment Accessibility and Education, covering Great Britain, developed by the Urban Big Data Centre (UBDC). The transport Dataset offer public transport availability (PTA) indicators at both the stop/station and small-area levels (lower layer super output area (LSOA) and middle layer super output area (MSOA)). The employment dataset provides information on the number of people with access to employment within specific distances from each output area. The housing datasets comprises of quarterly house rent and sales prices aggregated at output area level (MSOA). The education data comprises of secondary school (Greater Glasgow Area, Scotland) and Higher Education (Great Britain) student-level data. In addition to all educational outcomes at school stages S4-S6, the secondary school pupil data consists of age, gender, nationality and ethnic background, level of English, attendance, post-school destinations, and receipt of Gaelic education. This is augmented by individual schools’ data consisting of staffing levels, proportions of pupils’ speaking particular languages at home, religious denomination, distance travelled by students from home, and accessibility to greenspace from both the home and school neighbourhoods. The higher education (HE) dataset comprises of home and term-time locations (at postcode sector level), subject studied, level and mode of study of courses, level and classification of qualification, and post-HE destination. The theoretical background for measuring the datasets at small area levels is also presented in this paper. Additionally, a variety of raw data used to produce some of the datasets (e.g. PTA) is also introduced to enable interested readers to reproduce them.

**Specifications Table**

| **Subject area** | Social Science, Urban Studies, Transport Studies, Employment, Education, Housing |
| --- | --- |
| **More specific subject area** | Urban Area Analytics, Public transport services, Employment Access, Education Inequality Assessment, Housing Affordability |
| **Type of data** | CSV and Shapefiles |
| **How data was acquired** | Commercial listings, Survey, UK census data, UK Ordnance Survey data, Public Transport Schedule Data, and Office of National Statistics |
| **Data format** | Aggregated, Anonymized, Synthetic |
| **Experimental factors** | The transport dataset was transformed from TransXchange Format to general transit feed specification (GTFS), API was used to retrieve the housing data before being reprocessed, travel to work from UK Data Service’s Flow Data portal was linked to output area spatial boundaries using the geocodes. |
| **Experimental features** | New metrics were calculated based on a combination of different data sources. The GTFS data was subsequently used to create the PTA metrics at LSOA and MSOA levels. Census and travel to work datasets from UK Data Service’s Flow Data portal were used to create employment access metrics. Housing metrics were computed from Zoopla housing listings and education metrics were derived from datasets provided by Scottish Exchange of Data (ScotXed) and Higher Education Statistics Agency (HESA) data. |
| **Data source location** | Great Britain, except for Secondary School Data, from eight Scottish local authorities (Glasgow City, East and West Dunbartonshire, North and South Lanarkshire, Renfrewshire, East Renfrewshire, and Inverclyde). |
| **Data accessibility** | Some of the datasets that are publicly sharable are included with this article |

Value of the data

- Data offers country-wide urban area metrics (public transport availability (PTA), Housing, Employment access and Education) at small-area levels as well as stop/station-level (for PTA, based on service frequency and service area)
- The new urban area metrics can be used to study spatial and social inequalities in various facets of the urban areas (transport access, rental market dynamics, access to jobs, educational deprivation), and further estimate health, job, and educational outcomes of populations living in deprived areas (e.g. poor public transport services) see Anejionu et al., (in press).
- The data can also be used to compare impacts of policies, industrial and structural changes on intra-city dynamics across the entire country
- Data offers increased frequency of assessing and tracking changes in critical aspects of the urban area (housing rent prices fluctuations, spatial inequalities in PTA etc.) compared to decennial census or national survey datasets
- Longitudinal datasets can be used for in monitoring intra- and inter- annual spatiotemporal changes in the urban area with high level of spatial precision

# Data

## Transport Data

The transport data offer public transport availability indicators at both the stop/station and small area levels across Great Britain (England, Wales and Scotland). Specifically, we offer stop-level public transport availability data “GB_STOP_PTAI_2016.csv”, “GB_STOP_PTAI_2016.shp”, LSOA-level public transport availability data “GB_LSOA_PTAI_2016.csv”, “GB_LSOA_PTAI_2016.shp”, and MSOA-level public transport availability data “GB_MSOA_PTAI_2016.csv” and GB_MSOA_PTAI_2016.shp.

Tables 1 shows the number of observations in the public transport availability datasets at both the stop/station and small area levels across Great Britain. Figure 1 shows the distribution of stop-level PTAI by public transport service type. Table 2 shows small area geographies for different regions across Great Britain. Figure 2 shows distribution of MSOA-level PTAI for regions of GB. Scotland has a higher median of MSOA-level PTAI than other British regions.

Table 1. Description of public transport availability data at stop/station and small area levels

| **Data** | **Count of observations** | **Data table name** |
| --- | --- | --- |
| Stop-level public transport availability | 33,461 | GB_STOP_PTAI_2016 |
| LSOA-level public transport availability | 41,729 | GB_LSOA_PTAI_2016 |
| MSOA-level public transport availability | 8,480 | GB_MSOA_PTAI_2016 |


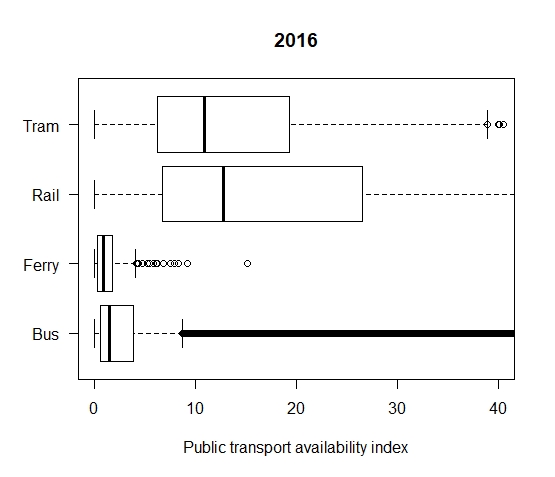


**Figure 1.** Distribution of stop-level PTAI by public transport service type, 2016

**Table 2.** Description of small area geographies for different regions

| Region | Count of MSOAs | Count of LSOAs | Population |
| --- | --- | --- | --- |
| North East | 340 | 1,656 | 2,635,506 |
| North West | 924 | 4,497 | 7,203,775 |
| Yorkshire and the Humber | 692 | 3,318 | 5,411,495 |
| East Midlands | 573 | 2,774 | 4,719,430 |
| West Midlands | 735 | 3,487 | 5,783,410 |
| East of England | 736 | 3,614 | 6,119,230 |
| London | 983 | 4,835 | 8,770,860 |
| South East | 1108 | 5,382 | 9,004,501 |
| South West | 700 | 3,281 | 5,508,645 |
| Wales | 410 | 1,909 | 3,113,150 |
| Scotland | 1,279 | 6,976 | 5,404,700 |


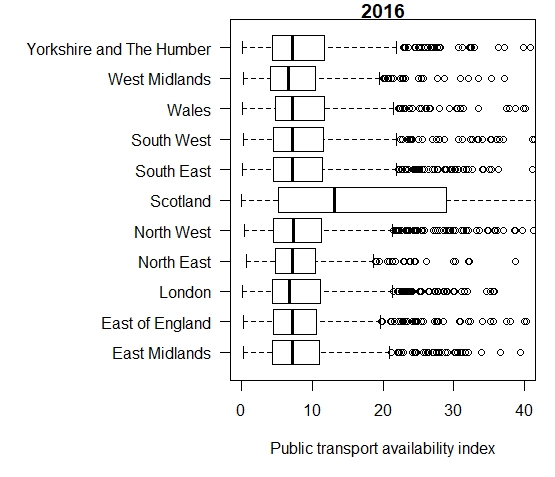


**Figure 2.** Distribution of MSOA-level PTAI for regions of GB, 2016

## Employment Access Data

This data offers the number of people within specific distances (5km, 10km, 20km, 25km, 30km, 40km, 50 km, 75km, 100km) from each output area with access to employment. This data is provided in this article in two formats: CSV (GB_Employment_Access.csv) and shapefile (GB_Employment_Access.shp).

## Education Data

The secondary education data comprises individual student data, at school stages S4-S6, from the eight local authorities making up the Greater Glasgow Area, and summary data about each secondary school, for each academic year from 2006/7 to 2014/5. In addition to all educational outcomes at SCQF levels 3-7, by subject, educational stage and product type, the information about students includes: age, gender, nationality and ethnic background, level of English, attendance, post-school destinations, distance to school, and accessibility to greenspaces from the home and school. Annual information about the schools is available for the same period, covering school staffing levels, and the proportions of pupils’ speaking particular languages at home.

The Higher Education dataset contains information on individual students for the academic years 2000/1-2015/6, cover the entire UK. Among the variables included are: home and term-time locations (at postcode sector level), subject studied, level and mode of study of courses, level and classification of qualification, and post-HE destination.

Both these datasets are considered very sensitive, and so they are held securely and cannot be shared with unauthorized persons.

## Housing Data

The housing data is an aggregated derivative of a data product acquired under license from *Zoopla Property Group (ZPG) Ltd.* It comprises of counts of number of advertisements for rental properties and properties for sale, current and historic median rent and sales of over 27 million residential property records across Great Britain, aggregated at MSOA levels and Broad Rental Market Areas (BRMA). across Great Britain at quarterly intervals.

This is a safeguarded data that cannot be shared openly due to legal conditions attached to the license by the data provider. However, it can be shared with registered non-commercial researchers, for a certain period. Aggregate data tables are available from UBDC website for personal use only. Individual researchers can access property level data for academic, non-commercial research use if they sign up to a corresponding end-user licence agreement. Interested researchers can contact UBDC directly to access this data.

# Experimental Design, Materials and Methods

## Great Britain’s small-area geography levels

In the UK demographic datasets, lower layer super output area (LSOA) and middle layer super output area (MSOA) are the two main small-area geography levels. MSOAs are built from groups of contiguous LSOAs. Typically, the average population of MSOAs is 7,200; while that of LSOAs is 1,500. There are now 34,753 LSOAs and 7,201 MSOAs in England and Wales (Office for National Statistics, 2015a). Scotland has independent demographic surveys and uses different names to represent the two small area geography levels. Scottish counterparts of MSOA and LSOA are intermediate zone (IZ) and data zone (DZ). Compared to England and Wales, Scotland is less densely populated. Therefore, IZ and DZ have larger areas but smaller population than MSOA and LSOA respectively. The population of MSOAs is 2,500 - 6,000; while that of DZ is 500 to 1,000. We merge English and Wales LSOA boundaries with Scottish DZ boundaries into a dataset “GB_LSOA_2011”, and merge English and Wales MSOA boundaries with Scottish IZ boundaries into a dataset “GB_MSOA_2011”.

Data provided in this project are aggregated to these small-area geographies as a way to anonymise them and to make them linkable to other socioeconomic datasets usually presented at these geographic levels.

## Transport Availability Index/Metrics

We propose a metric – transport availability index (PTAI) – to represent the levels of public transport service provisions at both stop/station and small area levels. Stop-level PTAI was measured by using public transport schedule data and stop/station location data. This was subsequently aggregated to small-area levels (LSOA and MSOA) in order to ensure PTAI is linkable to socioeconomic data at the same geography level. Specifically, stop-level PTAI was first aggregated to LSOA-level PTAI by overlaying service areas of stops/stations with LSOA boundaries. This was further aggregated to MSOA-level PTAI by weighting LSOA’s PTAI with its population. Data sources for this including the LSOA boundaries, MSOA boundaries and LSOA-level population are shown in Table 3.

**Table 3.** Data sources for the raw data used

| Data | Source |
| --- | --- |
| Non-train public transport service schedules | Traveline Information Limited, 2016a |
| Train service schedules | Network Rail Infrastructure Limited, 2014 |
| Locations of stops/stations | Traveline Information Limited, 2016b |
| Road network | Ordnance Survey, 2017 |
| English and Wales LSOA boundaries | Pope, 2017 |
| Scottish DZ Boundaries | Data.gov.uk, 2014a |
| English and Wales MOSA boundaries | Office for National Statistics, 2016 |
| Scottish IZ Boundaries | Data.gov.uk, 2014b |
| English and Wales LSOA-level population | Office for National Statistics, 2017 |
| Scottish DZ-level population | National Records of Scotland, 2017 |

### Public transport schedule data and stop/station location data

Raw public transport service schedule data of GB is offered by UK Traveline Information Limited and UK Network Rail Infrastructure Limited. More specifically, schedule data of non-train services (bus, light rail, tram, and ferry services) is stored in the TransXchange format, called the ‘Traveline National Dataset (TNDS)’ (Traveline Information Limited, 2016a); whilst schedule data of train services is stored in the common interface format (CIF) format, called ‘GB Rail Network’ (Network Rail Infrastructure Limited, 2014). Compared to TransXchange or CIF, general transit feed specification (GTFS) is a readable and widely used format for public transport schedule data. GTFS data of train services is available (Rail Delivery Group, 2016). However, for schedule data of non-train services were converted from TransXchange to GTFS via a Python conversion tool modified by the Urban Big Data Centre (UBDC) on the basis of an existing conversion tool (Mooney, 2016). This was spatially activated by combined it with stop/station location data offered by UK Traveline Information Limited (Traveline Information Limited, 2016b).

The train and non-train schedule datasets collected in July 2016 were combined into one dataset (“GB_GTFS_2016”) by the UBDC as pilot to demonstrate the generation of this new form of data for accessing public transport availability. Figure 3 shows the data processing in detail. Based on the GTFS schedule dataset and the stop/station location data collected in October 2016 (“GB_Stop_Location_2016”), 329,314 bus stops, 2,514 rail stations, 1,325 tram stations, and 306 ferry stations in operation across GB were used. This is in addition to 17,880 bus routes, 5,770 rail routes, 93 tram routes and 139 ferry routes in operation.


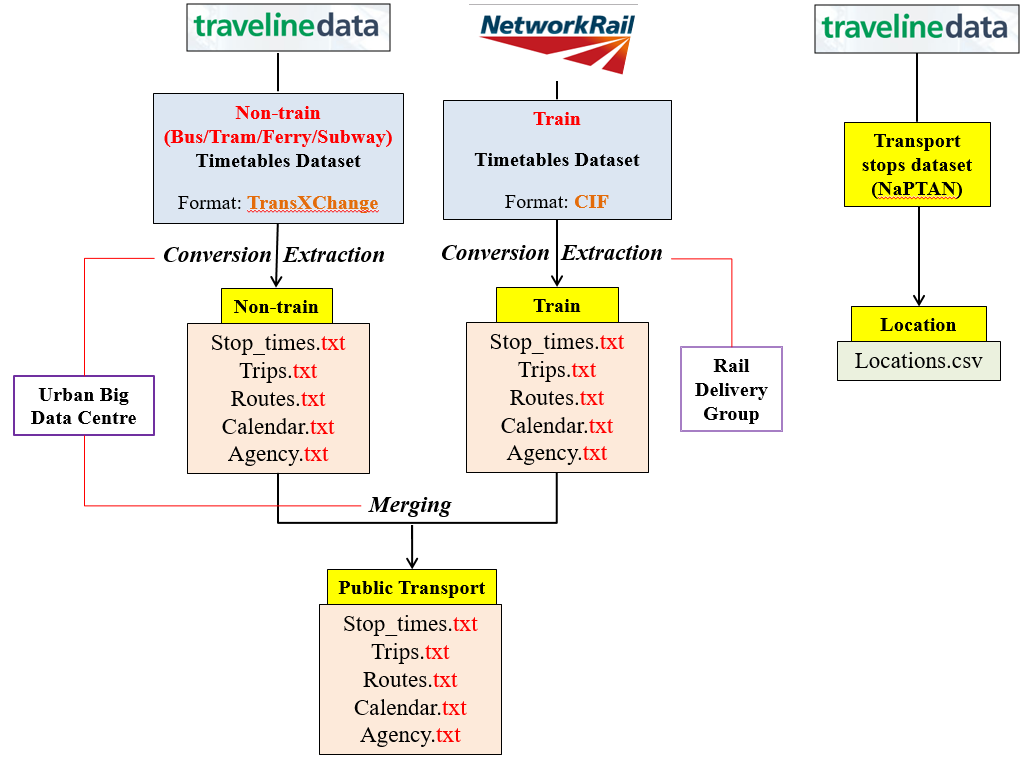


**Figure 3.** Public transport service schedule data conversion process

### Stop-level PTAI

To comprehensively measure levels of public transport availability, we take account of service frequency and service area as some studies proposed (Minocha et al., 2008; Currie, 2010; Delbosc and Currie, 2011). Moreover, we used an hour-weighted PTAI to represent public transport availability at the stop/station level. Identical service frequency in different daily time periods might influence accessibility for residents differently (e.g., peak time vs off-peak time). Service frequency in peak times seem to play a larger role than that in off-peak times (Minocha et al., 2008). Specifically, we determine the weights of hourly periods according to hourly distribution of trips in England as Scottish and Wales equivalents are not available. Weights of service hours are proportional to the number of trips in progress within hours as we assumed that high demand of trips within an hour means high importance of the hour. The UK National Travel Survey offers hourly number of trips in progress on weekdays (Monday to Friday) in England for 2015 (GOV.UK, 2013).

As public transport service schedules differ from weekdays and weekend days, we used only public transport services on weekdays rather than the entire week to measure public transport availability. This is reflective of the fact that vast majority of the residents’ journeys to basic destinations such as workplaces and schools occur mostly on weekdays. Hence, the PTA computed here measures how public transport service provisions support basic activities of local residents. Stop-level PTAI was computed as the weighted hourly number of trips passing a stop or station from Monday to Friday. Suppose *i* is a stop/station, its weighted PTAI is calculated as

$$Weighted\_PTAI(i)=\frac{1}{5}\sum_{t \in T} cnt\_trip\left( i,t \right) *w\left( t \right) (1)$$

where $cnt\_trip \left( i,t \right)$ is the total count of trips passing through the stop (station) *i* during the one-hour period *t* on the five working days, and $T$ is the set of one-hour periods.

LSOA-level PTAI

To accurately and comprehensively measure PTAI at the LSOA level, we took account of both the service levels and service areas of stations/stops. The service area is the area within which people are willing to walk to the station/stop along the road network. The desire to use public transport services declines as walking distance to a bus stop or a train station increases (Langford et al., 2012). Some studies reveal acceptable maximum walking distances differ from one public transport mode to another (Currie, 2010; Delbosc and Currie, 2011; Langford et al., 2012). A travel survey uncovers that 75% to 80% of people would access a stop/station if their walking distances are no longer than mode-specific acceptable maximum walking distances (Kittelson and Associates et al., 2003):

- Acceptable maximum walking distance to bus stop = 400 m.
- Acceptable maximum walking distance to tram stop = 400 m.
- Acceptable maximum walking distance to rail station = 800 m.
- Acceptable maximum walking distance to ferry station = 800 m.

A spatial buffer is used to represent service area of station/stop using the respective acceptable maximum walking distances. A circular buffer around the stops/station (Traveline Information Limited, 2016b; see Table 3), and road network buffer, based on the UK Ordnance Survey road network dataset covering Great Britain (Ordnance Survey, 2017; see Table 3) were used to generate service areas of stations/stops across GB.

Subsequently, stop-level PTAI were aggregated to LSOA by overlapping service areas of stations/stops with LSOAs. Figure 4 illustrates this, where LSOA *a* is served by Stop 1, Stop 2, Stop 3, Stop 4 and Station 1. For simplicity, regularly shaped buffers (circular buffers) were used to represent irregularly shaped buffers (road network buffers). Part of *a* is not served by any stop/station; while some areas of *a* are served by more than one stop/station. Suppose *L* is a LSOA, its PTAI is calculated as:

$PTAI(L)= \sum_{i\in S(L)} Weighted\_PTAI(i)*$ $\frac{Area (i \cap L)}{Area (L)}$ (2)

where *i* represents a stations/stop, and $S(L)$ is the set of stations/stops whose buffers intersect *L*. $Area (i\cap L)$ represents the overlapping area between *i* and *L*; and $Area (L)$ is the area of *L*.


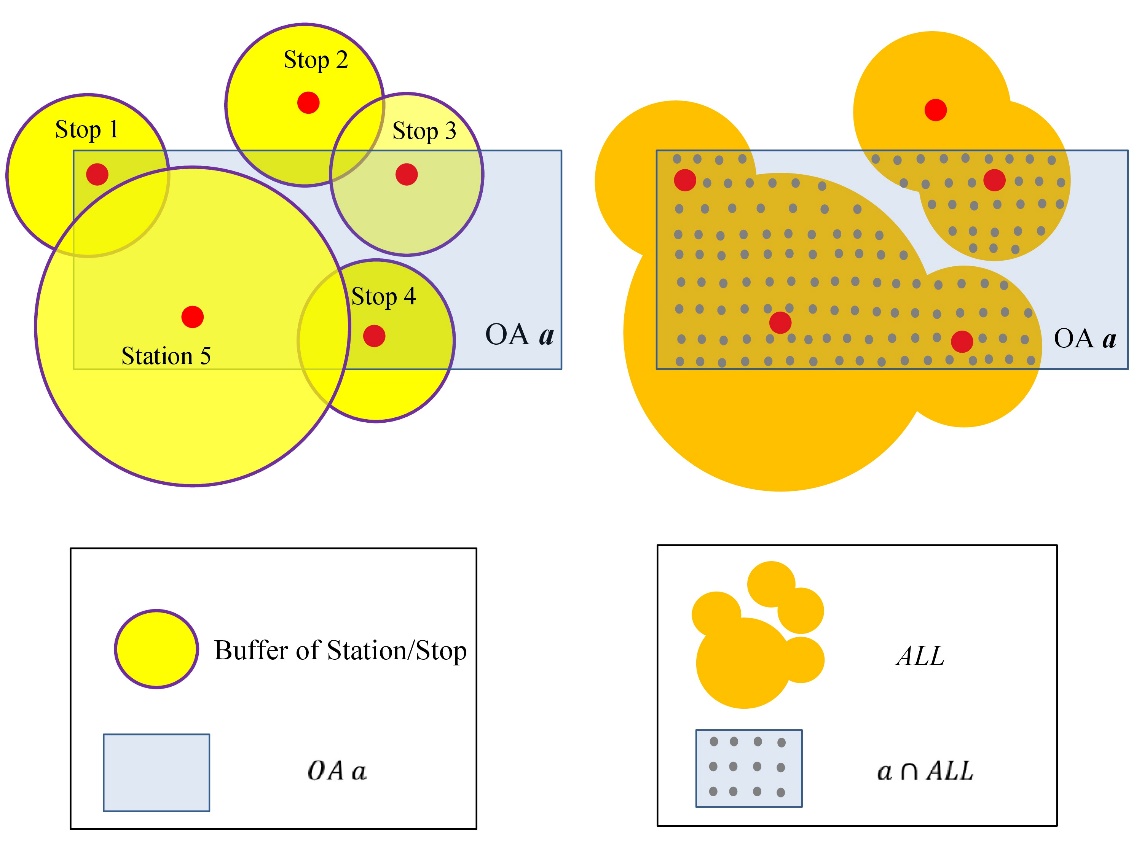


Figure 4: Simplified example of aggregating stop-level PTAI to LSOAs

### MSOA-level PTAI

Population-weighted PTAI was calculated at the MSOA level. Specifically, we aggregated LSOA-level PTAIs to MSOA by weighting LSOA’s PTAI with its population. Suppose *M* is a MSOA, its PTAI is calculated as:

$PTAI\left( M \right)= \sum_{j\in S\left( M \right)} PTAI\left( j \right)*$ $\frac{POP(j)}{POP(M)}$ (3)

where *j* represents a LSOA, and $S(M)$ is the set of LSOAs within *M*. $POP (j)$ is the population of LSOA *j*; and $POP (M)$ is the population of LSOA *M*.

## Employment Accessibility Metrics (EAM)

The need to continuously access more detailed geographical estimates of jobs and locations of workers at small-area levels over time at quarterly, and/or annual intervals motivated the generation of employment accessibility indicators in this project. This is an improvement compared to those currently available from the census or the Office of National Statistics (ONS), which are either aggregated at higher geographic levels (coarser detail) or are available only once every 10 years (decennial). The EAM is expected to enhance the understanding of the performance of different types of jobs (e.g., low-wage jobs or those in the service sector), as the economic dynamics (expansions, recessions or stagnation) changes.

### Generation of EAM

The number of people reporting that they worked in each output area (proxy for employment) was derived from travel to work data (2011 census), obtained from the UK Data Service’s Flow Data portal. The location of people’s residence and work (excluding quasi-workplaces) at the level of output area for the UK, was obtained from Table WF03UK_oa (https://wicid.ukdataservice.ac.uk/). Subsequently, the level of employment in each output area was estimated by aggregating the data by workplace output area. These employment data, combined with travel time information derived from the OpenStreetMap, were used to generate a number of labour market accessibility measures (Figure 5), using the gravity-based measure of potential accessibility developed by Hansen (1959). A measure of the cost of travelling between each pair of origins and destinations was required in this calculation. Distance along the road network was used as the measure of travel cost. The road network was represented using OpenStreetMap. An all-pairs shortest-path algorithm was then used to estimate a distance matrix.

Different methods have been developed to measure accessibility. A popular gravity-based method developed by Hansen (1959) was used to measure accessibility:

$A_{i}= \Sigma D_{j}f(c_{ij})$ (4)

where $A_{i}$is the accessibility index for zone i, $D_{j}$is a measure of the opportunities available at destination j, $c_{ij}$is the cost of travel between zones i and j, and f() is a cost deterrence function which reflects how distance affects the accessibility of opportunities. Here, D was used to represent the number of people stating they worked in each output area and $c_{ij}$ will be the network distance between output areas i and j.

The deterrence function was determined using a simple threshold function of the form:

$f(c_{ij}) = {\{}_{0 if c_{ij}> \tau}^{1 if c_{ij}\leq\tau}$ (5)

We evaluated the function for different levels of the parameter $\tau$. The accessibility measure gives the number of employment opportunities that can be reached within a given distance. One advantage of this measure is that it is easy to interpret.


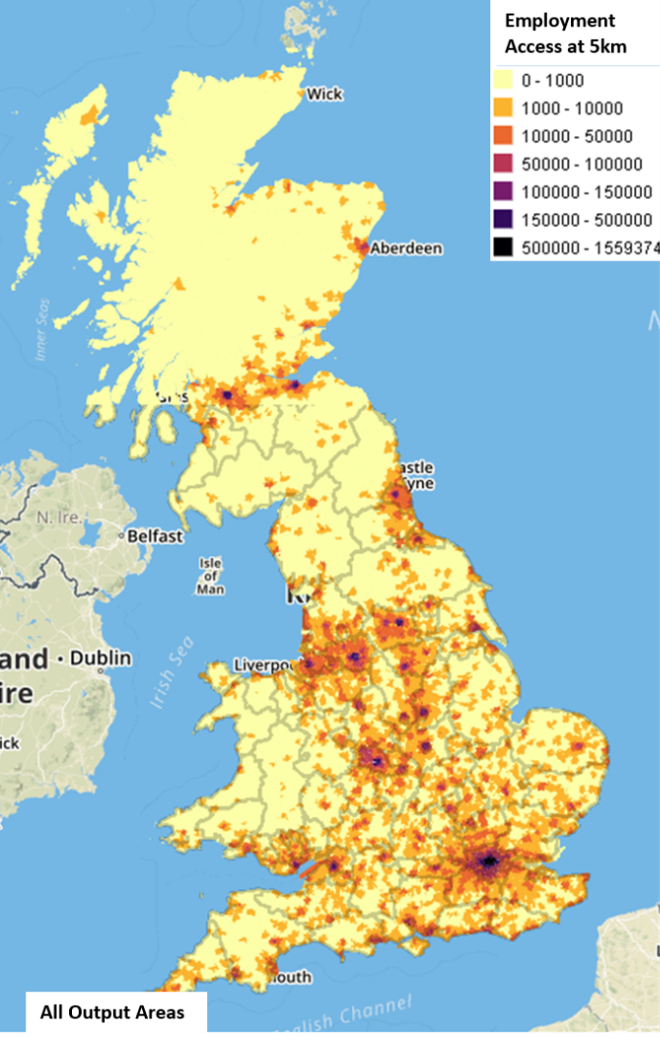


**Figure 5**. Maps showing employment opportunities within 5km (access 5km) of for all output areas across the GB.

## Housing Affordability Metrics (HAM)

Housing indicators are used to highlight the most important features of housing markets (Flood, 1997). The generation of Housing Affordability Metrics (HAM) in this project was motivated by the considerable knowledge gap concerning the scale and nature of housing dynamics, especially in the UK private-rented sector. The private rented sector is the most dynamic part of the UK housing system, having doubled in size in the last two decades, due to a number of factors including limited mortgage availability and diminished social housing. However, there is little data available to describe the sector (Office for Statistics Regulation, 2017). This gap emanates from the fact that most of the available information comes from survey data and decennial census data. Survey data tells a broad story at national, regional and local authority levels, and the UK Valuation Office Agency publishes rent tables to local authority level too. UK Census data offers higher spatial resolution but limited details about the sector. The available data resources are poor at representing lower geographies. This undermines a clear understanding of changes in the sector and associated issues, by local authorities, central government and researchers. Hence, to undertake continuous monitoring of the sector over time, housing market information has to be obtained from alternative sources. Data from Zoopla (<https://developer.zoopla.co.uk/>), a house listings aggregation service was considered a suitable alternative source for this crucial information. Our aggregate data product derived from the Zoopla property listings website offer additional spatial resolution (at MSOA, BRMA and Local Authority levels), providing details of numbers of adverts and mean/median rents per month by quarter for the period 2011-16. A historical dataset, available for academic, non-commercial research use under EULA terms provides wide-ranging insights about not only the rental and for-sale housing markets but also location, property features and property type within several fields including free text property descriptions and links to associated multimedia content. These have clear and obvious applications for housing researchers but may also be of interest to other urban studies disciplines, or as a corpus or basis for domain application for other data science work, such as text and linguistic analysis.

### Zoopla Data

Zoopla has over 27 million residential property records in their archive although only a relatively small percentage of these have been advertised for sale or rental on the Zoopla website and therefore contain a property listing history. Zoopla provides access to these historic property listings via an Application Programming Interface (API - https://developer.zoopla.co.uk/docs). UBDC has a licence to access this API with agreement to download data for the UK as part of the Centre's housing data catalogue. Housing data from properties advertised for sale or rent across Great Britain, from 2010 till present, were acquired, and complemented by price paid data (for sales) from the Land Registry of England and Wales and Registers of Scotland.

Baseline property listings (which contain various types of important historical information about properties) comprising 8 million property records (5 million advertised for sale and 3 million for rent) across Great Britain were initially generated via the Zoopla API with FME data extraction, transformation and loading (ETL) tool, and continuously updated as more properties left the market (closed listings). This has yielded a historic database for GB with over 5 million records of properties advertised for sale and 3 million records of properties advertised for rent. Nightly data collections from Zoopla’s live listings API (since August 2016) complement this historical dataset. Full UK coverage is available from 2010 with selected areas from as early as 2005. Figure 6 shows the number of adverts by sales or rental for 2010 – 2016, the initial period of historical data collection.

**Figure 6.** The number of Zoopla adverts by sales and rental for 2010 – 2016

### API processing

The API request to retrieve individual Zoopla property data requires unique property id. The daily active Zoopla property listings (current listings), which UBDC has been collecting since August 2016 include the property id value. However, for historical data (closed listings), not covered in the active listings, a method of accessing the complete Zoopla property id list for GB was required. The solution was to use the Zoopla estimates API. The requests to this API can use place names, postcode areas or user defined bounding boxes and the results returned include all property id's in that area.

The processing of the baseline property listings history is then comprised of the following steps:

1. Make a Zoopla estimates API request by area.
2. Extract individual property ids from the estimates results set.
3. Make a Zoopla property listing history API request for each property id extracted.

### Housing Metrics

A selection of aggregated data tables (Tables 4 and 5) comprising of count of rental adverts per quarter, mean and median rent per month per quarter for Local Authority, Broad Rental Market Area and Middle Super Output Area geographies were produced from the historical dataset. Aggregation to higher geographies was based on postcode so those listings with incomplete postcode information are excluded. Although these tables are available to download with no cost, usage is restricted to non-commercial reference only.

Table 4: Mean & Median Rent Per Month Per Quarter by Local Authority / BRMA / MSOA

| **Variable Name** | **Description** |
| --- | --- |
| authority_code / area_code | Spatial unit unique identifier |
| authority_name / brma_name | Spatial unit name |
| year | 4 digit year (2011 – 2016) |
| quarter | Quarter of year (1 – 4) |
| mean_rent_per_month | GBP mean rent |
| median_rent_per_month | GBP median rent |

Table 5: Count of Rental Adverts by Local Authority / BRMA / MSOA

| **Variable Name** | **Description** |
| --- | --- |
| authority_code / area_code | Spatial unit unique identifier |
| authority_name / brma_name | Spatial unit name |
| year | 4 digit year (2011 – 2016) |
| quarter | Quarter of year (1 – 4) |
| num_adverts | Total count of rental adverts |

To generate the housing affordability metrics, relevant housing attributes such as property IDs, address, price, description, date of advert, category, number of floors, were extracted from the Zoopla dataset. The data were linked to the LSOA spatial boundaries through the postcodes. Following this, aggregate data for key statistics (mean, median, maximum price, minimum for the rent and sale prices) of the properties, were computed at LSOA level (Figure 7).


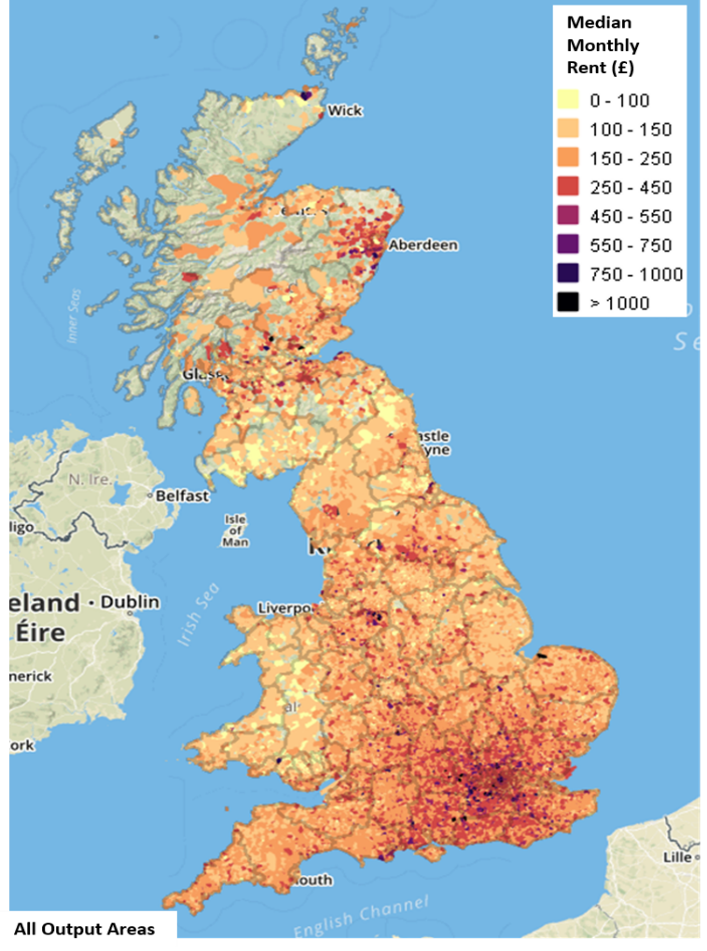


**Figure 7**. Maps showing monthly median rent price for all output areas across the GB.

## Education-Related Metrics

The desire to examine small-area-based drivers of inequalities in educational outcomes (Bell, 2003; Kerr et al, 2014), from Secondary School, through Further and Higher Education and into employment motivated the interest to create education-related metrics (ERM). This interest was also encouraged by Scotland’s Attainment Challenge (https://education.gov.scot/improvement/learning-resources/Scottish Attainment Challenge), launched in 2015 by the Scottish Government to achieve equity of educational opportunity and reduce poverty-related “attainment gap”. The metrics were derived from secondary school education data covering some parts of Scotland and from Higher Education data for the entire UK.

### Secondary School data

Secondary school data covering eight local authorities in the Glasgow City Region including: Glasgow City, East and West Dunbartonshire, North and South Lanarkshire, Renfrewshire, East Renfrewshire, and Inverclyde, were obtained from the Scottish Exchange of Data (ScotXed - http://www.gov.scot/Topics/Statistics/ScotXed). The datasets contain individual student-level data from the pupil census and data on all 31 publicly funded secondary schools for the academic years 2007/8 to 2015/16. Pupil data consisted of age, gender, nationality, ethnic background, level of English, receipt of Gaelic education, attendance, and post-school destinations. Educational attainment was measured for all units and courses at levels S4-S6 (senior secondary education, typically of those aged 14-17 years). Schools data covered staffing levels, religious denomination (in effect, multi/non-denominational vs. Roman Catholic) and proportions of pupils’ speaking particular languages at home. The linked pupil and school datasets were further extended with other derived and administrative data: the Euclidean distance travelled by students between home and school, and accessibility to greenspaces from the home and school neighbourhoods (determined from postcode centroids), and home and school locations were linked at datazone level (LSOA equivalent) to assign measures of deprivation and rurality.

### Higher Education Data

A fairly similar data for Higher Education dataset was generated from data supplied by the Higher Education Statistics Agency (HESA- https://www.hesa.ac.uk/). This is a secured data obtained through electronic Data Research and Innovation Service (eDRIS) special licencing arrangement (Safe Haven). It holds over 44m records for all students attending a Higher Education institution in the UK between 2000/1 and 2015/16, comprising personal characteristics (including home location at postcode sector level), and subject, level and mode of study of courses pursued, level and classification of qualification, and post-HE destination.

The Secondary School and Higher Education were subsequently used to develop the education metrics as a measure of student- and institution-based educational disadvantage at these stages of the educational career.

# Acknowledgements

We acknowledge the Economic and Social Research Council (ESRC) who funded the Urban Big Data Centre (UBDC) to undertake this project as part of the Big Data Phase 2 of the UK Research and Innovation.

- Data is Zoopla Limited, © 2018

**References**

Anejionu, O.C.D., Thakuriah, P.V., McHugh, A., Sun, Y., McArthur, D., Mason, P. and Walpole, R. (in press). Spatial Urban Data System: A Cloud-enabled Big Data Infrastructure for Social and Economic Urban Analytics. Future Generation Computer Systems(FGCS).

Bell, J. (2003). Beyond the school gates: The influence of neighbourhood on the relative progress of pupils. *Oxford Review of Education*, 29, 485-502.

Currie, G., 2010. Quantifying spatial gaps in public transport supply based on social needs. J. Transp. Geogr. 18, 31-41.

Data.gov.uk, 2014a. Data Zone Boundaries 2011. <https://data.gov.uk/dataset/ab9f1f20-3b7f-4efa-9bd2-239acf63b540/data-zone-boundaries-2011>

Data.gov.uk, 2014b. Intermediate Zone Boundaries 2011. <https://data.gov.uk/dataset/133d4983-c57d-4ded-bc59-390c962ea280/intermediate-zone-boundaries-2011>

Delbosc, A., Currie, G., 2011. Using Lorenz curves to assess public transport equity. J. Transp. Geogr. 19(6), 1252-1259.

Flood, J. (1997). Urban and Housing Indicators. Urban Studies, 34, (10), 1635-1665.

GOV.UK, 2013. National Travel Survey: When people travel. https://www.gov.uk/government/statistical-data-sets/nts05-trips

Hansen, W.G. (1959) How Accessibility Shapes Land Use, Journal of the American Institute of Planners, 25:2, 73-76, DOI: 10.1080/01944365908978307

Kerr, K., Dyson, A., Raffo, C. (2014). Education, Disadvantage and Place: Making the Local Matter, Bristol: Policy Press.

Kittelson & Associates, KFH Group, Parsons Brinkerhoff Quade and Douglas Inc., & JHunter-Zaworski, K., 2003. Transit Capacity and Quality of Service Manual, second ed. Transit Cooperative Research Program TCRP, Washington, DC.

Langford, M., Higgs, G., Fry, R., 2012. Using floating catchment analysis (FCA) techniques to examine intra-urban variations in accessibility to public transport opportunities: the example of Cardiff, Wales. J. Transp. Geogr. 25, 1-14.

Minocha, I., Sriraj, P.S., Metaxatos, P., Thakuriah, V., 2008. Analysis of transit quality of service and employment accessibility for the greater Chicago, Illinois, region. Transp. Res. Record 2042, 20-29.

Mooney, A., 2016. txc2gtfs: Python tool to convert TransXChange data to GTFS. <https://github.com/adamlukemooney/txc2gtfs>

National Records of Scotland, 2017. Mid-2016 Small Area Population Estimates for 2011 Data Zones. <https://www.nrscotland.gov.uk/statistics-and-data/statistics/statistics-by-theme/population/population-estimates/2011-based-special-area-population-estimates/small-area-population-estimates/mid-2016/detailed-data-zone-tables>

Network Rail Infrastructure Limited, 2014. GB rail network. <https://datafeeds.networkrail.co.uk/ntrod/login>

Ordnance Survey, 2017. OS Open Roads. <https://www.ordnancesurvey.co.uk/business-and-government/products/os-open-roads.html>

Office for National Statistics, 2015a. Census geography. <https://www.ons.gov.uk/methodology/geography/ukgeographies/censusgeography>

Office for National Statistics, 2016. Middle Layer Super Output Areas (December 2011) Full Clipped Boundaries in England and Wales. <http://geoportal.statistics.gov.uk/datasets/middle-layer-super-output-areas-december-2011-full-clipped-boundaries-in-england-and-wales>

Office for National Statistics, 2017. Lower Super Output Area Mid-2016 Population Estimates. <https://www.ons.gov.uk/peoplepopulationandcommunity/populationandmigration/populationestimates/datasets/lowersuperoutputareamidyearpopulationestimates>

Office for Statistics Regulation (2017) Statistics on housing and planning in the UK: systematic review of public value. London: UK Statistics Authority

Pope, A., 2017. 2011 lower layer super output areas (LSOA) boundary, [Dataset]. University of Edinburgh. <http://dx.doi.org/10.7488/ds/1896>.

Rail Delivery Group, 2016. Rail Industry Data. <http://data.atoc.org>

Traveline Information Limited, 2016a. Traveline Open Data: Traveline National Dataset. <http://www.travelinedata.org.uk/traveline-open-data/traveline-national-dataset>

Traveline Information Limited, 2016b. Transport stops (NaPTAN): NaPTAN dataset (National Public Transport Access Nodes). https://www.travelinedata.org.uk/other-transport-open-data/transport-stops

1. Corresponding author: Obinna C.D. Anejionu. obinna.anejionu@glasgow.ac.uk [↑](#footnote-ref-2)
